# Rapid and Precise Computation of Fractional Flow Reserve from Routine Two-Dimensional Coronary Angiograms Based on Fluid Mechanics: The Pilot FFR2D Study

**DOI:** 10.3390/jcm13133831

**Published:** 2024-06-29

**Authors:** Grigorios G. Tsigkas, George C. Bourantas, Athanasios Moulias, Grigorios V. Karamasis, Fivos V. Bekiris, Periklis Davlouros, Konstantinos Katsanos

**Affiliations:** 1 Department of Cardiology, University Hospital of Patras, 26504 Patras, Greece; dramoulias@live.com (A.M.); pdav@upatras.gr (P.D.); 2 Medlytic Labs, 26222 Patras, Greece; georgios.bourantas@gmail.com (G.C.B.); fbekiris@gmail.com (F.V.B.); katsanos@med.upatras.gr (K.K.); 3 Second Cardiology Department, Attikon University Hospital, National and Kapodistrian University of Athens Medical School, Rimini 1, Chaidari, 12462 Athens, Greece; grigoris.karamasis@gmail.com; 4 Department of Interventional Radiology, School of Medicine, University of Patras, 26222 Patras, Greece

**Keywords:** fractional flow reserve, two-dimensional, coronary angiography, precision, accuracy

## Abstract

**Objective:** To present a novel pipeline for rapid and precise computation of fractional flow reserve from an analysis of routine two-dimensional coronary angiograms based on fluid mechanics equations (FFR2D). **Material and methods:** This was a pilot analytical study that was designed to assess the diagnostic performance of FFR2D versus the gold standard of FFR (threshold ≤ 0.80) measured with a pressure wire for the physiological assessment of intermediate coronary artery stenoses. In a single academic center, consecutive patients referred for diagnostic coronary angiography and potential revascularization between 1 September 2020 and 1 September 2022 were screened for eligibility. Routine two-dimensional angiograms at optimal viewing angles with minimal overlap and/or foreshortening were segmented semi-automatically to derive the vascular geometry of intermediate coronary lesions, and nonlinear pressure–flow mathematical relationships were applied to compute FFR2D. **Results:** Some 88 consecutive patients with a single intermediate coronary artery lesion were analyzed (LAD n = 74, RCA n = 9 and LCX n = 5; percent diameter stenosis of 45.7 ± 11.0%). The computed FFR2D was on average 0.821 ± 0.048 and correlated well with invasive FFR (r = 0.68, *p* < 0.001). There was very good agreement between FFR2D and invasive-wire FFR with minimal measurement bias (mean difference: 0.000 ± 0.048). The overall accuracy of FFR2D for diagnosing a critical epicardial artery stenosis was 90.9% (80 cases classified correctly out of 88 in total). FFR2D identified 24 true positives, 56 true negatives, 4 false positives, and 4 false negatives and predicted FFR ≤ 0.80 with a sensitivity of 85.7%, specificity of 93.3%, positive likelihood ratio of 13.0, and negative likelihood ratio of 0.15. FFR2D had a significantly better discriminatory capacity (area under the ROC curve: 0.95 [95% CI: 0.91–0.99]) compared to 50%DS on 2D-QCA (area under the ROC curve: 0.70 [95% CI: 0.59–0.82]; *p* = 0.0001) in predicting wire FFR ≤ 0.80. The median time of image analysis was 2 min and the median time of computation of the FFR2D results was 0.1 s. **Conclusion:** FFR2D may rapidly derive a precise image-based metric of fractional flow reserve with high diagnostic accuracy based on a single two-dimensional coronary angiogram.

## 1. Introduction

Coronary angiography allows for evaluation of the anatomical and morphological characteristics of stenoses of vascular geometries but is not accurate in identifying functionally significant coronary artery stenoses—also known as ‘critical’ stenoses—in as many as one third (1/3) of the cases [1]. Intracoronary techniques using dedicated pressure wires have been developed to measure physiological indices as surrogates of inducible myocardial ischemia. Amongst many, fractional flow reserve (FFR) has emerged as the leading modality to guide revascularization and improve clinical outcomes in patients with stable coronary artery disease (CAD) and non-ST-elevation acute coronary syndromes (ACS) [2]. However, wire-based methods for FFR measurement are inherently invasive and may suffer from pitfalls and limitations limiting their use and widespread adoption [3]. Current FFR utilization rates in assessing intermediate coronary artery stenoses vary by geography and remain low at generally less than 10% of the relevant cases [4].

Contrary to invasive wire-based methods, image-based methods have been recently developed to study the physiology of coronary flow and pressure and derive mathematical approximations of FFR and other indices without the use of pressure wire instruments. Computer models of blood pressures and coronary flows range from one-dimensional mathematical models to three-dimensional mesh models employing computational fluid dynamics based on Navier–Stokes formulas [5,6]. However, computer models are complex, often time-consuming, and universally rely on the analysis of three-dimensional images that require extra acquisition, analytical, and computational steps. The authors herein propose a novel non-invasive image-based computer pipeline that may rapidly derive a quantitative analysis of the physiological significance of intermediate coronary stenoses using two-dimensional angiographic acquisitions (FFR2D) obtained during routine coronary angiography. Computation of FFR2D is governed by fluid mechanics equations and may process standard angiograms from optimal projection angles depending on the vessel of interest. This is a pilot analytical study that was designed to assess the diagnostic performance of FFR2D versus standard two-dimensional quantitative coronary angiography (2DQCA; % diameter stenosis ≥ 50%) to predict FFR ≤ 0.80 for the identification of physiologically significant lesions using the gold standard of FFR measurements with a pressure wire.

## 2. Material and Methods

### 2.1. Study Population

This was a post-hoc observational analysis from a single high-volume academic center. Consecutive patients referred for diagnostic coronary angiography and potential revascularization between 1 September 2020 and 1 September 2022 were screened for eligibility based on pre-set inclusion and exclusion criteria, as outlined in Table 1. The study was reviewed by the local ethics committee, and the need for informed consent was waived because this was a retrospective analysis of fully anonymized patient images without any deviation from standard of care. The anonymized image datasets were transferred and processed retrospectively off-site on a dedicated desktop computer (Medlytic Labs S.A.) by an operator masked to the actual FFR recording. Wire-based FFR was considered as the gold standard reference test for physiological evaluation of coronary lesions. The FFR cut-off value for identification of a flow-limiting stenosis was ≤0.80 for all lesions per the guideline-recommended standard of care [7].

### 2.2. Invasive FFR Measurement

Any commercial FFR vendor was allowed, according to the experience and preference of the operator per standard of care for guiding the decision to intervene on coronary artery lesions. The pressure wire was prepared, calibrated, and equalized according to the instructions for use. Routine use of intravenous adenosine as a vasodilator was applied per local policy via a peripheral vein infusion at 140 μg/kg/min. The entire FFR interrogation study was recorded and FFR tracings were available for retrospective analysis to exclude aortic damping or distortion of the waveform or pressure drift after wire pullback. FFR was calculated as the ratio of distal mean pressure (Pd) measured by the pressure wire to the proximal mean pressure (Pa) during hyperemia. The lowest recorded value of the captured FFR trace was accepted as the reference test. The FFR cut-off for identification of a flow-limiting stenosis was ≤ 0.80 for all lesions. The wire location was documented angiographically for all measurements. Quality control of the FFR recordings used for analysis was performed by two interventional cardiologists with more than 5 years of experience in FFR measurement.

### 2.3. Fluid Mechanics Framework

Contrary to complex computational fluid dynamics techniques, we have developed the FFR2D mathematical model based on the non-linear mathematical formulas governing pressure–flow relationships in stenosed arteries under low-resistance hyperemic conditions. In addition, we computed FFR based on image analysis of routine two-dimensional angiographic acquisitions obviating the need for a more demanding method with reconstruction of three-dimensional geometries that mandate acquisition and processing of two or more views at different projection angles.

The hemodynamic severity of arterial stenoses is governed by a non-linear relationship between pressure and flow that was first studied by Gould et al. and Young et al. in the 1970s [8,9]. Briefly, the reduction of pressure (and hence flow) that occurs across an arterial stenosis is non-linear with respect to its geometrical severity (percent of reduction of luminal cross-sectional diameter or area), and, typically, negligible pressure drops are detected unless a critical stenosis of at least 50% diameter reduction has developed. With progressive arterial narrowing, coronary autoregulation will compensate for pressure loss and flow reduction at resting conditions until 85% diameter reduction [10]. However, pressure drops with a non-linear relationship with blood flow velocity, and thereby the physiological significance of a given stenosis is accentuated at higher flow rates. Kirkeeide et al. have long documented that the cross-sectional shape of the stenosis (circular or oval or otherwise irregular) has no apparent effect on expansion pressure drop [11]. Pressure loss depends strongly and predominantly on stenosis severity, which is best expressed as area reduction and not diameter reduction, regardless of the asymmetry or eccentricity of its cross-sectional shape [12]. It has been established that pressure loss across arterial stenosis is well-described by quadratic equations that describe pressure drops as the sum of viscous, turbulent, and inertia contributions [13,14]. Vascular resistance *R* and pressure drop Δ*p* are governed by the following Equations (1) and (2), respectively, where the linear coefficient *k*_1_ governs the energy loss due to viscous friction, and the quadratic coefficient *k*_2_ determines the energy loss due to flow turbulence and separation [8,9].

(1) $$R=\frac{\Delta p}{Q}=k_{1}+k_{2}Q$$

and

(2) $$\Delta p=k_{1}Q+k_{2}Q^{2}$$

Young and Tsai have elaborated on this in relation to blood velocity *U* (13) by defining pressure drop, as shown in Equation (3).

(3) $$\Delta p=\frac{k_{v}\mu }{D}U+\frac{k_{t}}{2}{\left(\frac{A_{0}}{A_{1}}-1\right)}^{2}\rho \left|U\right|U+k_{u}\rho L\frac{dU}{dt}$$

Based on this theoretical background, FFR2D can quickly calculate the fractional flow reserve from a single two-dimensional angiographic run without using any vasodilator agents. The novel FFR2D© method for computing pressure losses across one or more arterial stenoses and deriving fractional flow reserve (FFR) in a coronary artery without a vasodilator briefly includes the following steps:

1. Measuring the mean arterial pressure (MAP) in a coronary artery inlet and regressing a hyperemic MAP based on the literature (Pa).
2. Acquiring a two-dimensional angiography image and delineating the vascular contours to compute the vessel geometric characteristics.
3. Measuring the time taken for blood to flow across the interrogated vessel segment and calculating the volumetric flow rate of the blood vessel.
4. Inferring a state of maximum hyperemia based on the regression of experimental data.
5. Calculating a pressure drop Δ*p* across each of the interrogated artery stenoses by adapting the Gould et al. [8] and Young et al. [9] formulas to derive a mean intra-coronary pressure Pd at the distal end of the stenosis.
6. Improving precision of computed Δ*p* by k-fold cross-validation analysis of hyperemic and resting pressure measurements.
7. Calculating fractional flow reserve FFR2D based on the simple formula of FFR2D = Pd/Pa.

The whole pipeline is patient-specific, as it uses a combination of anatomical, morphological, and hemodynamic information. Vessel volume across the measured artery segment is approximated under an axisymmetric assumption. The volumetric flow rate of the analyzed coronary geometry is approximated with the well-known TIMI frame count (TFC) method that captures the velocity of the injected contrast agent within the interrogated coronary artery over a measured vessel length and time [15].

### 2.4. Outcomes and Statistics

The primary study hypothesis was that FFR2D has good correlation with the invasive FFR reference test and superior diagnostic performance compared to two-dimensional quantitative coronary angiography (2DQCA; % diameter stenosis ≥ 50%) to predict FFR ≤ 0.80 for the identification of physiologically significant lesions. Secondary endpoints included the feasibility rate of FFR2D computation from a single-view angiographic image in the FFR-interrogated vessels, bias, precision of FFR2D, and time of image processing and computation for calculation of the FFR2D results.

Categorical baseline patient demographics, clinical variables, and angiographic vessel characteristics are presented as count and percentages. Continuous variables are presented as means and standard deviation if originating from a normal distribution or reported as medians and inter-quartile range if non-normally distributed. The baseline clinical variables were analyzed on a per-patient basis and the angiographic lesion characteristics and FFR results on a per-vessel basis. An FFR2D value ≤ 0.80 is scored as positive implying a hemodynamically critical stenosis, whereas an FFR2D value > 0.8 is negative and would defer revascularization. The FFR2D diagnostic accuracy was assessed with the area under the curve (AUC) of the receiver-operating characteristic (ROC) analysis using invasive FFR as the reference test. A cut-off value of FFR ≤ 0.80 was applied to identify functional stenoses. The vessel-based AUCs for the FFR2D and 2DQCA models were compared with the non-parametric DeLong test [16].

By taking FFR as the reference solution, we report diagnostic classification indexes including accuracy (Acc), sensitivity (Sen), specificity (Spe), positive predictive value (PPV), and negative predictive value (NPV) for the computed FFR2D values to detect a physiologically significant stenosis on the cut-off value of ≤0.80. The false negative ratio and false positive ratio are reported along with likelihood ratios. All diagnostic outcomes are dichotomously scored per target vessel in comparison with invasive FFR. The Pearson correlation coefficient r was computed between FFR2D and invasive FFR. A Bland–Altman analysis with 95% agreement levels was performed to present the agreement between the FFR2D and FFR references. Statistical analyses were performed with Graphpad Prism version 8.0.1 and Statsdirect version 3.3.5. A two-sided value of *p* < 0.05 was applied to determine statistical significance.

## 3. Results

### 3.1. Patient Cohort

**Figure 1:**
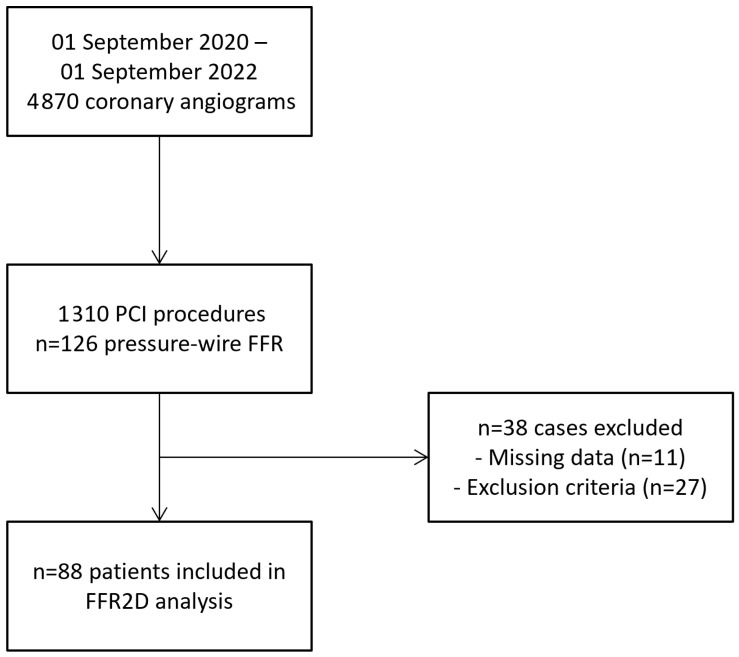
Study flowchart shows inclusion of 88 consecutive patients eligible for FFR2D computation.

Some 88 consecutive patients with single intermediate coronary artery lesions were successfully analyzed. Figure 1 shows the flowchart of the study—13 cases were excluded because of clinical, and 14 according to angiographic, exclusion criteria—mainly ostial or diffuse lesions. The latter included three vessels with severe tortuosity/foreshortening and/or vessel overlap precluding accurate image analysis for FFR2D computation. Therefore, the overall feasibility of successful FFR2D computation was 88 out of 91 (96.7%). The baseline variables and clinical characteristics of the patients were typical of chronic coronary artery disease and are outlined in Table 2. Lesions were located primarily in the left anterior descending artery (LAD; n = 74), with nine cases in the right coronary artery (RCA) and five in the left circumflex artery (LCX). Radial access and peripheral intravenous infusion of adenosine was applied in all cases. Lesions were located at coronary bifurcations in 22 cases (25.0%), and tandem stenoses were found in 9 arteries (10.2%; n = 5 with two lesions and n = 4 with three lesions). Quantitative coronary angiographic analysis documented a minimum lumen diameter of 1.76 ± 0.48 mm with an average percent (%) diameter stenosis of 45.7 ± 11.0%. With the TIMI frame count method, the average contrast velocity was 14.2 ± 5.9 cm/s, and the volumetric flow rate was 0.83 ± 0.53 mL/s at resting conditions.

### 3.2. Diagnostic Performance

**Figure 2:**
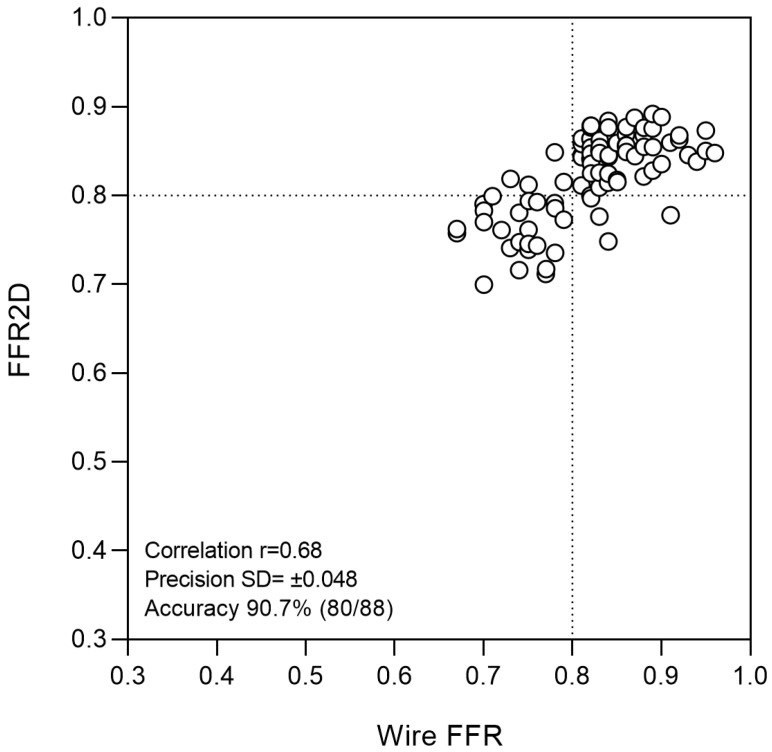
Correlation of FFR2D and wire FFR was good (r = 0.68; *p* < 0.001), with an overall 90.7% accuracy of correct prediction (80 out of 88 cases). Measurement bias was minimal, with a mean difference between FFR2D and wire FFR of 0.000 ± 0.048.

**Figure 3:**
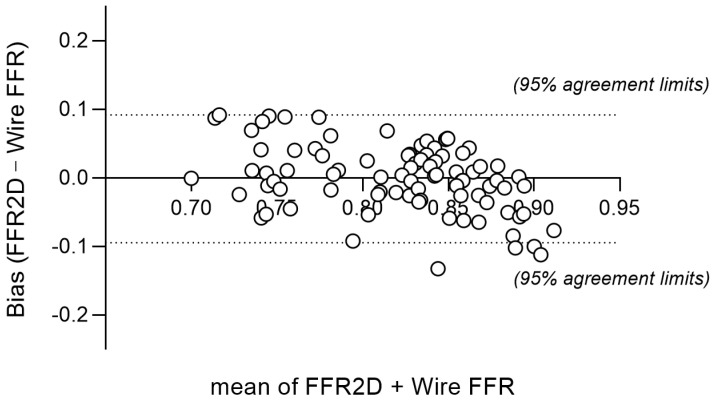
Bland–Altman plot shows very good agreement of FFR2D and wire-FFR; dashed lines denote the mean difference ± 1.96SD ranging from −0.096 to +0.096. There was minimal bias of 0.000 ± 0.049.

**Figure 4:**
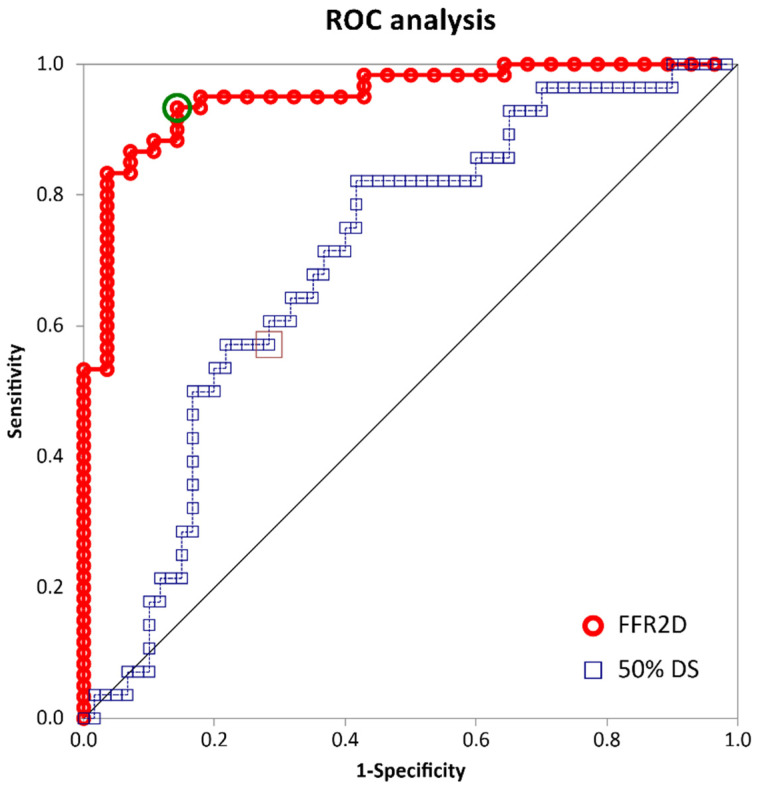
ROC plots. Red line denotes the ROC curve of FFR2D, and blue line denotes the ROC curve of 50%DS on 2D-QCA in identifying physiologically significant coronary artery stenoses based on the criterion of pressure wire FFR ≤ 0.80.

The mean arterial pressure at rest was 93.9 ± 7.0 mm Hg, and the measured pressure wire FFR was 0.821 ± 0.067 (median 0.826). Abnormal FFR ≤ 0.80 was measured in 28 cases (31.8%). The computed FFR2D was on average 0.821 ± 0.048 (median 0.837) and correlated well with invasive FFR (r = 0.68, *p* < 0.001). There was a very good agreement between FFR2D and invasive wire FFR with minimal bias (mean difference: 0.000 ± 0.048)—Figure 2 and Figure 3. Applying the FFR cut-off value of ≤ 0.80 to FFR2D resulted in 24 true positives, 56 true negatives, 4 false positives, and 4 false negatives. FFR2D ≤ 0.80 predicted the measured FFR ≤ 0.80 with a sensitivity of 85.7%, specificity of 93.3%, positive likelihood ratio of 12.7, and negative likelihood ratio of 0.15. The overall diagnostic accuracy of FFR2D for diagnosing a critical epicardial artery stenosis as defined by FFR ≤ 0.80 was 90.9% (80 cases classified correctly out of 88 in total). A summary of all diagnostic statistics with their corresponding 95% confidence intervals is included in Table 3, which compares the results to 2D-QCA on a 50% diameter stenosis threshold. FFR2D had superior discriminatory capacity compared to percent diameter stenosis at the 50% threshold in diagnosing physiologically significant intermediate coronary artery stenoses using the FFR cut-off value of ≤0.80. On ROC analysis, FFR2D had a significantly higher area under the curve (0.95 [95% CI: 0.91–0.99]) than 50%DS on 2D-QCA (0.70 [95% CI: 0.59–0.82]; *p* = 0.0001). The corresponding ROC curves are shown in Figure 4. The median time of image analysis was 2 min, and the median time of computation of the FFR2D results was 0.1 s.

### 3.3. Subgroup Analyses

Lesions were located at coronary bifurcations in 22 cases (25.0%), and arteries with tandem lesions were analyzed in 9 cases. In bifurcational lesions (n = 22), the measurement bias was −0.011 ± 0.051, and sensitivity, specificity, and overall diagnostic accuracy were 100%, 89.4%, and 90.9%, respectively. In the case of tandem lesions (n = 9), the measurement bias was −0.004 ± 0.040, and sensitivity, specificity, and overall diagnostic accuracy were 100%, 100%, and 100%, respectively. Considering FFR cases close to the 0.80 threshold (values ranging between 0.77 and 0.83) where classification certainty was around 80% for repeated measurements [17], the diagnostic performance of FFR2D was evaluated separately within and outside the FFR grey zone by excluding the latter values. Within the wire FFR grey zone (n = 31 cases), the sensitivity, specificity, and overall diagnostic accuracy of FFR2D were 75.0%, 87%, and 83.9%, respectively. Outside the wire FFR grey zone (n = 55 cases), the sensitivity, specificity, and overall diagnostic accuracy of FFR2D were 90.0%, 97.3%, and 94.7%, respectively.

## 4. Discussion

The authors have developed a novel method for rapid and precise computation of fractional flow reserve from routine two-dimensional coronary angiograms (FFR2D) based on a pipeline of fluid mechanics. Contrary to complex and time-consuming computational fluid dynamics techniques, the present FFR2D mathematical model is based on the non-linear mathematical formulas governing pressure–flow relationships in stenosed arteries under low-resistance hyperemic conditions [8,9]. In addition, FFR is computed based on image analysis of routine two-dimensional angiographic acquisitions obviating the need for arduous reconstruction of three-dimensional geometries that mandate acquisition and processing of at least two or more views at different projection angles. The present analysis has shown that FFR2D is an easily applicable method that may rapidly derive the physiological parameters of interest with a high probability of correct classification of critical epicardial stenoses (ROC area under the curve: 0.95). Overall, the proposed method was easy to implement through routine two-dimensional QCA analysis. It was characterized by very low image exclusion rate and superior discriminatory capacity in identifying critical stenoses compared to routine QCA only, rapid sub-second computation times, and, most importantly, high precision measurements compared to wire FFR.

The concept of studying the pressure–flow relationship under hyperemic conditions to define a critical threshold of hemodynamic significance for the treatment of coronary arteries was first introduced by Pijls et al. in 1993 [18]. Fractional flow reserve (FFR) is a hemodynamic index that quantifies the functional severity of a coronary artery stenosis and is defined as the maximum achievable blood flow in the presence of a stenosis divided by maximum flow in the absence of any obstructive epicardial coronary disease at peak hyperemia. However, Young et al. first described that the physiological significance of a stenosis cannot be judged solely based on its geometrical characteristics but rather in association to given flow conditions [13,19]. Under resting conditions, autoregulation helps to maintain normal flow and oxygen delivery to the tissues peripheral to the stenosis by simply reducing the distal microvascular resistance. However, as the stenosis increases and breaches a certain critical threshold, distal flow will be reduced dramatically because peripheral resistance cannot be further reduced to offset the large pressure drop across the stenosis [9,11]. Along those same principles, the mathematical framework of FFR2D is innovative in computing volumetric flow rates and deriving pressure drops across healthy and stenosed coronary segments—both at rest and at simulated hyperemia—and thereby enabling the rapid physiological assessment of intermediate coronary artery lesions with neither the application of invasive FFR instrumentation nor uncomfortable use of adenosine.

Current guidelines recommend the use of FFR in identifying functionally significant epicardial coronary artery stenoses and determining the need for PCI. However, wire-based methods for FFR measurement suffer from several pitfalls and limitations restricting their use and applicability to a minority of eligible cases. First, they mandate the administration of pharmacological vasodilators that produce patient discomfort during injection. Second, wire-based FFR increases the duration of the procedure, and is inherently invasive and costly to implement. In addition, poor handling performance of the pressure guidewires may render difficulties and complications during wire manipulation across complex lesions. Finally, almost one-third of FFR tracings suffer from either significant drift or waveform abnormalities, diminishing the quality of the measurements [3]. To avoid the discomfort of pharmacological hyperemia, several invasive wire-based methods that measure non-hyperemic pressure ratios (NHPR) have been developed (e.g., DFR, RFR, iFR, etc.). All resting pressure-derived indices correlate well with each other and generally demonstrate similar diagnostic performance in examining lesions and deferring the need for treatment [20]. Comparative analysis of NHPR measurements from 1129 cases against hyperemic pressure wire FFR showed that NHPR (e.g., iFR) had wide ±0.17 limits of agreement (with a biased FFR + 0.09 mean estimate) that would often change clinical management decisions [21]. Hence, discordances between hyperemic FFR and NHPR are encountered in more than 1 out of 5 cases, significantly limiting the reliability of the latter.

Of note, the herein reported accuracy, sensitivity, and specificity of FFR computation from routine two-dimensional coronary angiograms appear to numerically exceed the diagnostic performance of invasive non-hyperemic indices, and the authors believe that image-based approaches like FFR2D may not only obviate the need for invasive pressure wires but also prove more accurate than non-hyperemic pressure ratios in predicting cardiac ischemia defined by FFR ≤ 0.80. In fact, the FFR2D approach allows for holistic assessment of coronary physiology, including the direct measurement of axisymmetric vessel volume, contrast transit time, blood velocity and volumetric flow rates, and pressure losses along the vessel at resting conditions and the subsequent determination of pressure drops during simulated hyperemic conditions that are non-linearly related to vessel geometry. Theoretically, with the same equations, one may also infer basal and hyperemic stenosis resistance, as well as basal and hyperemic microvascular resistance.

There is already some software commercially available as a medical device (SaMD) for image-based derivation of FFR aiming to provide non-invasive physiology guidance for disease screening or for use inside the catheterization laboratory. However, all of them require laborious reconstruction of three-dimensional anatomies as a first step, they universally suffer from significant exclusion rates, and they have so far demonstrated inferior diagnostic precision outcomes. The HeartFlow software is based on 3-D image reconstruction of coronary anatomy from thin-section cardiac computed tomography angiograms (CCTA) coupled with Navier–Stokes CFD formulas to approximate FFR in different arterial territories. However, the procedure takes several hours, and on average 10–15% of the studies are rejected because of image quality or high heart rate [22,23]. For example, in the prospective NXT pivotal trial, 13% of the CTA datasets were rejected by the core lab because of image artifacts precluding FFRCT analysis. In addition, there was on average a −0.03 ± 0.07 measurement error [24], with other independent studies reporting further lower precision (error standard deviation ranging from 0.12 to 0.14) [25]. The quantitative flow ratio (QFR) is an alternative functional index that is again based on 3-D image reconstruction from two separate orthogonal coronary angiograms employing virtual hyperemic conditions and faster FFR computations in the order of 5 min [26]. Diagnostic performance of QFR is in line with the herein-reported FFR2D results [27]; however, a systematic review of 2933 patients and 3335 vessels found that 18% of evaluated vessels could not be analyzed [28], and precision was average (error standard deviation 0.08) [25,27]. The Cathworks FFRAngio is another angio-based approach that mandates three different angiographic projection angles but claims a brief processing time of 2.7 min by employing a computational lump model based on scaling laws of vascular resistances and allows for physiological assessment of the whole left or right coronary tree. In a recent pooled analysis of prospective studies with 700 lesions from 588 patients, there was good precision (error standard deviation 0.06 to 0.07) [29,30]. However, the reported exclusion rate was nearly 7% in the FAST-FFR pivotal study, and data is lacking about real-world applicability of the technology.

Compared to the angiogram-based technologies, the herein-proposed FFR2D has a minimal exclusion rate as it utilizes routine 2-D angiographic acquisitions of sufficient quality for diagnostic purposes. Most importantly, it is associated with high measurement precision (standard deviation of measurement error was 0.048), which compares favorably to expected errors of wire-based FFR. To compare, in the case of repeated FFR measurements in the DEFER trial (Deferral Versus Performance of PTCA in Patients Without Documented Ischemia), the standard deviation of the measurement error was 0.032 [31]. In the recently published DEFINE-FLOW study, precision was similar, and standard deviation of the difference of repeated FFR measurements was 0.041. The authors therefore believe that the FFR2D measurement error is quite low and well within what would be expected from the normal variance of FFR measured with invasive pressure wires. In addition, if there is good contrast opacification in a single plane without severe tortuosity/foreshortening and/or vessel overlap, FFR2D is characterized by very low rejection rates, e.g., less than 3% in the present study.

There are several important limitations in the present study. First, image analysis to derive vascular geometry is semi-automated and involves some user interaction for the correction of contour detection. In addition, the user needs to select the first and last frame for the TIMI frame count method to determine coronary artery flow [15]. Both previous steps may introduce user heterogeneity and reproducibility bias. A 15-frame-per-second acquisition rate is therefore advocated, and the user needs to select a diastolic frame where the vessel and the stenosis present with the least foreshortening and overlap. Hence, adequate training and operator certification is needed. Second, contrary to 3-D vessel reconstructions, 2-D imaging may not accurately represent the true asymmetry or eccentricity of a given coronary artery stenosis [32]. Considering that minimal luminal cross-sectional area is the most critical determinant of the hemodynamic severity, we postulate that FFR2D may under- or overestimate pressure losses in cases of highly irregular lesions. Third, the FFR2D pipeline is patient-specific and incorporates volumetric flow rate across the interrogated vessel for derivation of pressure losses. Hence, its true performance remains uncertain in cases of bifurcational lesions, as well as arteries with significant tapering where the flow distribution and energy losses may deviate from experimental models [13,14]. Of further note, left circumflex and right coronary artery lesions were poorly represented in the present dataset. To the latter end, analysis of dedicated larger samples sizes is required. Admittedly, exclusion criteria, such as ostial or diffuse lesions, severe tortuosity, and vessel overlap, may restrict the applicability of image-based FFR software like FFR2D in real-world clinical scenarios. Fourth, this was a retrospective analysis of a consecutive patient cohort in a single academic center, and FFR2D computations were performed offline. Hence, the sample size was small, limiting the generalizability and robustness of the findings. In addition, single-center studies may be characterized by inherent bias related to local practices and patient demographics. Measurement precision and true exclusion rates are best investigated in a prospective pivotal multicenter study with on-site vessel analysis.

In conclusion, we have presented a novel method for rapid and precise computation of fractional flow reserve from routine two-dimensional coronary angiograms (FFR2D) based on a simple framework of fluid mechanics physics. The FFR2D technology is innovative as it obviates laborious 3-D vessel reconstructions, it skips the need for uncomfortable pharmacological agents to induce hyperemia, and it may predict FFR with sub-second mathematical computations and very low image exclusion rates, potentially enabling near-real-time incorporation into the cath lab throughput. Most importantly, it allows for the holistic assessment of cardiac physiology with a high probability of correct classification of critical epicardial stenoses and high precision measurements compared to either competing angio-based technologies or pressure wire measurements themselves. Further validation is required in larger multicenter prospective studies.

## Figures and Tables

**Table 1:** Study design.

**Inclusion Criteria:** Male or female subjects >18 years of age. Patients with stable angina or unstable angina or NSTEMI Use of wire based FFR to evaluate at least one culprit or non-culprit coronary artery stenosis. Intermediate coronary artery stenosis with 30–90 percent diameter stenosis (%DS) and a reference vessel diameter of at least 2 mm by visual estimate Administration of routine vasodilators (Adenosine or Papaverine) as hyperemic stimulus to derive physiological significance and decide the need for revascularization. Availability of at least one high-quality angiographic view of the vessel under interrogation **Clinical Exclusion Criteria:** Recent acute infarct (STEMI) or documented prior STEMI on same side (right/left). Prior CABG, heart transplant, or valve surgery, or prior TAVI/TAVR Severe heart failure (NYHA ≥ III)/alternatively known LVEF ≤ 45% **Angiographic Exclusion Criteria:** Ostial left main or ostial right coronary artery lesions Distal diffuse lesions in combination with proximal coronary lesions Poor contrast opacification or image panning during acquisition Poor image quality precluding contour detection Severe overlap of stenosed segments with other branches or collaterals Severe tortuosity or foreshortening of target coronary vessel Chronic total occlusions or target coronary vessel that is supplied by major collaterals

**Table 2:** Baseline clinical and angiographic variables.

Baseline Variables	Sample n = 88
Age (years) *	65.8 ± 10.6
Male gender	66/88 (75.0%)
Hyperlipidemia	68/88 (77.3%)
Smoking	59/88 (67.0%)
Diabetes	20/88 (22.7%)
Hypertension	73/88 (83.0%)
Family CAD history	13/88 (14.8%)
Previous PCI intervention	28/88 (31.8%)
Previous CABG surgery	0/88 (0.0%)
Asymptomatic positive stress test	50/88 (56.8%)
Stable angina	18/88 (20.5%)
Unstable angina	20/88 (22.7%)
Ejection fraction (%) *	53.2 ± 9.8
Radial access	88/88 (100%)
Adenosine intravenous	88/88 (100%)
Vessel bifurcations	27/88 (30.7%)
Vessel calcifications	24/88 (27.3%)
Tandem lesions	10/88 (11.4%)
Anatomy—index artery	
LAD	74/88 (84.1%)
RCA	9/88 (10.2%)
LCX	5/88 (5.7%)
Minimum lumen diameter *	1.76 ± 0.48 mm
Reference vessel diameter *	3.25 ± 0.56 mm
Percent (%) diameter stenosis *	45.7 ± 11.0%

* Reported as mean ± standard deviation.

**Table 3:** Diagnostic performance statistics.

Statistical Test	FFR2D (≤0.80)	2D-QCA (50%DS)
Disease prevalence	31.8 (22.3–42.6)%	31.8 (22.3–42.6)%
Sensitivity	85.7 (67.3–95.9)%	57.1 (37.2–75.5)%
Specificity	93.3 (83.8–98.2)%	71.7 (58.6–82.6)%
Accuracy	90.9 (82.9–96.0)%	67.1 (56.2–76.7)%
Positive predictive value	85.7 (69.7–94.0)%	48.5 (30.8–66.5)%
Negative predictive value	93.3 (84.9–97.2)%	78.2 (64.9–88.2)%
Positive likelihood ratio	13.0 (4.93–33.54)	2.02 (1.21–3.37)
Negative likelihood ratio	0.15 (0.06–0.38)	0.60 (0.38–0.94)
ROC area under the curve	0.95 (0.91–0.99)	0.70 (0.59–0.82)

## Data Availability

Data not publicly available due to proprietary analyses performed by Medlytic Labs.
